# Sensory Deprivation and Brain Plasticity

**DOI:** 10.1155/2012/810370

**Published:** 2012-10-09

**Authors:** Maurice Ptito, Ron Kupers, Steve Lomber, Pietro Pietrini

**Affiliations:** ^1^École d'Optométrie, Université de Montréal, Montréal, QC, Canada H3T 1P1; ^2^Department of Neuroscience and Pharmacology, Faculty of Health Sciences, University of Copenhagen, Copenhagen, Denmark; ^3^Danish Research Center for Magnetic Resonance, Hvidovre Hospital, DK-2650 Hvidovre, Denmark; ^4^Department of Physiology and Pharmacology, The University of Western Ontario, London, ON, Canada N6A5K8; ^5^Centre for Brain and Mind, Department of Psychology, The University of Western Ontario, London, ON, Canada N6A 5K8; ^6^Laboratory of Clinical Biochemistry and Molecular Biology, University of Pisa Medical School, 56126 Pisa, Italy

Early experience plays a key role in the development of the central nervous system as the brain has to adapt continuously to environmental changes that threaten its harmonious development. This adaptation phenomenon is called brain plasticity and refers to the lifelong changes in the structure of the brain that accompany experience (experience-dependent plasticity). Brain plasticity implies that the brain is pliable and can be remolded in response to challenges that interfere with its normal development such as sensory deprivation, brain injury, or abnormal development. Indeed, several regimens of visual deprivation such as dark rearing, enucleation, or eye-lid suturing lead to alterations of the visual system. Conversely, environmental enrichment also produces regional changes in brain anatomy such as increased dendritic space for synapses, increased cortical thickness, and elevated gene expression.

Sensory deprivation at birth or perinatally has dramatic effects on the organization of the brain circuitry, leading to a takeover of the deprived cortex by other sensory modalities. This phenomenon is known as cross-modal plasticity and is mediated by the development of new inter-modal connections or by strengthening or unmasking of already existing connections. Cross-modal plasticity has been demonstrated following visual and auditory deprivation since birth. In both cases, the deprived cortex becomes activated by one or more of the remaining senses, touch, audition, and olfaction in the case of blindness and visual stimulation in the case of deafness. There is a large body of evidence, based upon anatomical, metabolic, and behavioral findings, that the deprived sensory cortex acquires multiple sensory and cognitive functions, transforming itself therefore into a multimodal cortex. 

In this issue, we have gathered a number of review and original papers on the topic of brain plasticity in humans and in animal models of unimodal sensory deprivation. We have welcomed not only papers dealing with various sensory systems such as vision, audition, touch, and olfaction but also those dealing with basic mechanisms of brain plasticity. Several papers present results on cross-modal plasticity and sensory substitution in humans and in animal models of sensory deprivation. Using elegant methodologies, these studies highlight how other sensory modalities take over the deprived visual or auditory cortices in blind and deaf subjects, respectively. Brain plasticity can also be triggered by changes in sensory experience. Neural reorganization will take place if the environment is modified during the early stages of development as is the case following cortical lesions of the visual cortical areas, visual deprivation through eyelid suturing, or dark-rearing (M. W. Burke et al.; H. Bengoetxea et al.). As shown by J. F. Maya-Vetencourt and N. Origlia, visual cortex plasticity results from a complex interplay between the individual's genetic background and the environment. M. Pietrasanta and coworkers describe how the interhemispheric connectivity between the visual cortices can be altered by visual deprivation. The corpus callosum therefore seems to have a pivotal role in plasticity of the visual cortex. The papers by G. Dormal and colleagues and M. Ptito and colleagues provide evidence that the functional segregation of the efferent projections of the primary visual cortex into a dorsal and a ventral stream is preserved in congenitally blind humans. A. Leo and colleagues focus on the purported role of the parietooccipital connections in conveying nonvisual information to the deprived visual cortex. Two papers focus on auditory deprivation and cross-modal plasticity. M. A. Meredith and B. L. Allman show how the auditory cortex of deaf ferrets is reorganized by the somatosensory modality, whereas P. Hirst and colleagues use a primate model to highlight cross-modal plasticity. D. M. Coppola describes a model of olfactory system plasticity through unilateral naris occlusion. The author points to the role of the spared ipsilateral olfactory pathway in olfactory plastic responses and also discusses several methodological caveats.

The papers by K. Lehmann and colleagues and S. Desgent and M. Ptito address the role of GABAergic inhibitory interneurons in brain maturation and brain plasticity. These papers describe the important role of GABAergic inhibitory interneurons in maintaining the appropriate dynamic range of cortical excitation, in establishing critical periods of developmental plasticity, in receptive field refinement, and in the cortical processing of sensory information. This interneuron population is very sensitive to sensory experience during development, and visual deprivation studies emphasize the important contribution of these inhibitory interneurons in cross-modal plasticity.

We hope that this special issue will shed light on the various mechanisms involved in cross-modal plasticity and sensory substitution and contribute to the development of new tools that will boost training-induced plasticity. 

*Maurice Ptito* *Maurice Ptito* *Ron Kupers* *Ron Kupers* *Steve Lomber* *Steve Lomber* *Pietro Pietrini* *Pietro Pietrini*

